# Targeting of human interleukin-12B by small hairpin RNAs in xenografted psoriatic skin

**DOI:** 10.1186/1471-5945-11-5

**Published:** 2011-02-27

**Authors:** Rasmus O Bak, Karin Stenderup, Cecilia Rosada, Line B Petersen, Brian Moldt, Frederik Dagnæs-Hansen, Maria Jakobsen, Søren Kamp, Thomas G Jensen, Tomas N Dam, Jacob Giehm Mikkelsen

**Affiliations:** 1 Department of Human Genetics, University of Aarhus, Aarhus, Denmark; 2 Department of Dermatology, Aarhus University Hospital, Aarhus, Denmark; 3 Department of Medical Microbiology and Immunology, University of Aarhus, Aarhus, Denmark; 4 Department of Dermatology, Roskilde Hospital, Roskilde, Denmark

## Abstract

**Background:**

Psoriasis is a chronic inflammatory skin disorder that shows as erythematous and scaly lesions. The pathogenesis of psoriasis is driven by a dysregulation of the immune system which leads to an altered cytokine production. Proinflammatory cytokines that are up-regulated in psoriasis include tumor necrosis factor alpha (TNFα), interleukin-12 (IL-12), and IL-23 for which monoclonal antibodies have already been approved for clinical use. We have previously documented the therapeutic applicability of targeting TNFα mRNA for RNA interference-mediated down-regulation by anti-TNFα small hairpin RNAs (shRNAs) delivered by lentiviral vectors to xenografted psoriatic skin. The present report aims at targeting mRNA encoding the shared p40 subunit (IL-12B) of IL-12 and IL-23 by cellular transduction with lentiviral vectors encoding anti-IL12B shRNAs.

**Methods:**

Effective anti-IL12B shRNAs are identified among a panel of shRNAs by potency measurements in cultured cells. The efficiency and persistency of lentiviral gene delivery to xenografted human skin are investigated by bioluminescence analysis of skin treated with lentiviral vectors encoding the luciferase gene. shRNA-expressing lentiviral vectors are intradermally injected in xenografted psoriatic skin and the effects of the treatment evaluated by clinical psoriasis scoring, by measurements of epidermal thickness, and IL-12B mRNA levels.

**Results:**

Potent and persistent transgene expression following a single intradermal injection of lentiviral vectors in xenografted human skin is reported. Stable IL-12B mRNA knockdown and reduced epidermal thickness are achieved three weeks after treatment of xenografted psoriatic skin with lentivirus-encoded anti-IL12B shRNAs. These findings mimick the results obtained with anti-TNFα shRNAs but, in contrast to anti-TNFα treatment, anti-IL12B shRNAs do not ameliorate the psoriatic phenotype as evaluated by semi-quantitative clinical scoring and by immunohistological examination.

**Conclusions:**

Our studies consolidate the properties of lentiviral vectors as a tool for potent gene delivery and for evaluation of mRNA targets for anti-inflammatory therapy. However, in contrast to local anti-TNFα treatment, the therapeutic potential of targeting IL-12B at the RNA level in psoriasis is questioned.

## Background

Psoriasis is a chronic inflammatory skin disorder generally manifesting itself as symmetrical, erythematous, and scaling papules and plaques. The disease affects approximately 2-3% of the population worldwide and has a negative impact on the physical wellbeing and the quality of life [1-5]. Histologically, psoriasis displays epidermal hyperplasia, parakeratosis, thinning of stratum granulosum, and dilated and prominent vascularization of the dermis associated with an increased cellular infiltrate of immune cells. The exact cause of psoriasis is unknown, but it is widely accepted that a dysregulated immune system plays a pivotal role. Numerous pro-inflammatory cytokines are up-regulated in psoriasis and a normalization of the cytokine milieu has been shown to improve the disease phenotype [6-10]. For example, several inhibitors of tumor necrosis factor alpha (TNFα), which is considered one of the primary mediators of immune regulation, have been developed and proven successful in psoriasis treatment [11-13].

The pro-inflammatory cytokines interleukin-12 (IL-12) and IL-23 are both up-regulated in lesional psoriatic skin compared to non-lesional skin [14-17]. Both interleukins are expressed by activated denditric cells and macrophages present in the skin, but also to some extent by keratinocytes [17-19]. IL-12 stimulates the production of IFN-γ and the maturation of naïve T-cells into T_h_1 cells [20]. IL-23 seems to play a crucial role in the survival and proliferation of T_h_17 cells, leading to the production of IL-17 and in turn the pro-inflammatory cytokines TNFα, IL-1, IL-6, IL-8, and IL-22. Genetic polymorphisms in IL-12B and one of the IL-23 receptor subunits (IL-23R), have been linked to psoriasis [21], and many of the current therapies used in treating psoriasis, such as narrow-band UVB therapy [22] and administration of Etanercept (soluble TNFα receptor) [23] or Alefacept (an antagonist of T cell activation) [24] all reduce levels of IL-23. IL-12 and IL-23 are considered important factors in initiating and driving the T_h_1 and T_h_17 cytokine profiles characteristic of psoriasis.

IL-12 and IL-23 share a common subunit, the p40 subunit (encoded by the IL-12B gene) with the implication that both interleukins can be inhibited simultaneously. This therapeutic approach was recently validated with the approval of the p40-targeting monoclonal antibody, Ustekinumab, for clinical use [25]. In a phase III trial, Ustekinumab was shown to be more effective and requiring fewer injections than the TNFα-inhibitor Etanercept [26]. Although biological therapeutics inhibiting cytokines have proven successful in the treatment of moderate to severe psoriasis, there is still an unmet need for treatments that are convenient, without side-effects or contra-indications, and well tolerated, especially for long-term treatment. Of note, the biological therapeutics used today are administered systemically, where topically and locally administered treatments may be more desirable in terms of reducing systemic side-effects.

We have previously documented the therapeutic applicability of targeting TNFα mRNA by lentiviral delivery of anti-TNFα RNA effectors to xenografted psoriatic skin [8]. We tested here the hypothesis that targeting of IL-12B mRNA by RNA interference (RNAi)-mediated degradation is therapeutically relevant. RNAi is a natural cellular mechanism by which double-stranded RNAs (dsRNAs) are processed into ~21-nucleotide small interfering RNAs (siRNAs) which can mediate sequence-specific degradation of target RNAs [27]. If synthetic siRNA duplexes or DNA encoding small hairpin RNAs (shRNAs) are transfected into cells they are efficiently processed by the RNAi machinery and enter the RNAi pathway [28,29]. Thus, potent and specific down-regulation of a single gene can be achieved by siRNA/shRNA delivery, and this is already a widely established tool for gene suppression. shRNA delivery by human immunodeficiency virus-derived lentiviral vectors benefits from high transduction efficiency as well as specific and stable down-regulation of gene expression [30] due to the inherent property of lentiviruses to integrate the genetic cargo into the genome.

In the current report, we describe the establishment of a lentiviral vector platform for an easy one-step cloning of shRNA oligonucleotides. Employing this novel lentiviral vector, we screened a panel of shRNAs targeting different regions of IL-12B mRNA and identified a potent shRNA candidate which mediated efficient IL-12B mRNA knockdown in several *in vitro *assays. To investigate the therapeutic applicability of targeting IL-12B mRNA in psoriasis, we employed the psoriasis xenograft transplantation model [31]. Administration of the clinically approved p40-targeting antibody, Ustekinumab, was included as a comparative control to validate the relevance of targeting IL-12B in this model. Efficient lentiviral transgene delivery to xenografted psoriatic skin was demonstrated by intradermal injection of lentiviral vectors encoding a luciferase reporter gene, and stable and persistent luciferase expression was monitored for more than three months. Lentiviral delivery of anti-IL-12B shRNAs resulted in reduction of epidermal thickness and reduction in IL-12B mRNA levels. However, clinical evaluation of the psoriatic phenotype and immunohistochemical examination of the skin grafts did not indicate clinical resolution in this model. Taken together, this suggests that RNAi-directed targeting of IL-12B mRNA in lesional psoriatic skin is not as clinically potent as targeting of TNFα-encoding mRNA [8].

## Methods

### Contruction of plasmids

pCCL-cPPT-PGK-Puro-WPRE-LTR-H1-MCS (designated pCCL-PGK-Puro-H1-MCS) was constructed from pCCL-cPPT-PGK-puro-WPRE-LTR-H1-shTNFα [8] identical to the construct reported by Raoul *et al. *[32] except for the eGFP cassette being replaced by a puromycin resistance cassette and the shRNA targeting TNFα. By using two primer sets ((i) 5'-caccgtcaccgccgacgtcg-3' and 5'-ggcgcgcctcgctagcctcctagggtggtctcatacagaactta-3' and (ii) 5'-cctaggaggctagcgaggcgcgccgcggccgccaccgcggtgga-3' and 5'-cgctctagatgctgctagag-3'), two PCR fragments were amplified from this plasmid amplifying the two regions upstream and downstream of the shTNFα including the flanking XhoI and the XbaI sites, respectively. The two primers flanking the shTNFα region were made with restriction site containing linkers, so an overlap extension PCR between the two PCR fragments would replace the shTNFα with a MCS (AvrII, NheI and AscI). XhoI/XbaI digestion of the overlap PCR fragment allowed for insertion into the XhoI/XbaI-digested pCCL-cPPT-PGK-puro-WPRE-LTR-H1-shTNFα, replacing the shTNFα cassette with the MCS. For each of the shRNA expression constructs, pCCL-PGK-Puro-H1-shIL12B # 1-7, complementary sense and antisense oligonucleotides (see table 1) were annealed by incubation at 100°C for 5 minutes followed by room temperature cooling. The annealed oligonucleotides were designed to leave overhangs for cloning into the AvrII/AscI-digested pCCL-PGK-Puro-H1-MCS vector. For construction of psiCHECK-IL12B, a 1046-bp IL-12B cDNA was amplified from an IL-12B ORF (IMAGE ID: 30915173, Geneservice Ltd, Cambridge, UK) with primers 5'-aaactcgagccattggactctccgtcctg-3' and 5'-cctccaaattttcatcctgg 3'. The 3'UTR was amplified from cDNA from total RNA from human psoriatic skin using primers 5'-aggttctgatccaggatgaaaatttggagg-3' and 5'-aaagcggccgcgattacaaagaagagttttt-3'. Briefly, total RNA was purified with SV Total RNA Isolation System (Promega, Madison, WI, USA) and first-strand cDNA synthesis was performed with the AffinityScript™ QPCR cDNA Synthesis Kit (Stratagene, La Jolla, California, USA), both according to manufacturer's protocol. An overlap extension PCR with primers 5'-aaactcgagccattggactctccgtcctg-3' and 5'-aaagcggccgcgattacaaagaagagttttt-3' joined the two fragments, creating a 2336-bp IL-12B cDNA that was cloned into the psiCHECK-2 vector (Promega, Madison, WI, USA) using the XhoI/NotI sites.

**Table 1:** Oligonucleotides used for creation of anti-IL12B shRNA expression cassettes

shRNA	Oligonucleotide sequence (target sequences are underlined)
shIL12B #1	**Sense: 5'-ctagccccctcacctgtgacacccctactcgagaaggggtgtcacaggtgaggtttttggaaa-3**'
	**Antisense: 5'-cgcgtttccaaaaacctcacctgtgacaccccttctcgagtaggggtgtcacaggtgaggggg-3**'

shIL12B #2	**Sense: 5'-ctagcccatgccagcattagcgtgcgactcgagacgcacgctaatgctggcattttttggaaa-3**'
	**Antisense: 5'-cgcgtttccaaaaaatgccagcattagcgtgcgtctcgagtcgcacgctaatgctggcatggg-3**'

shIL12B #3	**Sense: 5'-ctagcccggtcttaggctctggcaaaactcgagatttgccagagcctaagacctttttggaaa-3**'
	**Antisense: 5'-cgcgtttccaaaaaggtcttaggctctggcaaatctcgagttttgccagagcctaagaccggg-3**'

shIL12B #4	**Sense: 5'-ctagcccggaatttggtccactgataactcgagatatcagtggaccaaattcctttttggaaa-3**'
	**Antisense: 5'-cgcgtttccaaaaaggaatttggtccactgatatctcgagttatcagtggaccaaattccggg-3**'

shIL12B #5	**Sense: 5'-ctagcccgcagacagatgcaaagaaaactcgagatttctttgcatctgtctgctttttggaaa-3**'
	**Antisense: 5'-cgcgtttccaaaaagcagacagatgcaaagaaatctcgagttttctttgcatctgtctgcggg-3**'

shIL12B #6	**Sense: 5'-ctagcccgatgggaacgcaagagatactactcgagaagtatctcttgcgttcccatctttttggaaa-3**'
	**Antisense: 5'-cgcgtttccaaaaagatgggaacgcaagagatacttctcgagtagtatctcttgcgttcccatcggg-3**'

shIL12B #7	**Sense: 5'-ctagcccgggccttcatgctatttaaactcgagattaaatagcatgaaggccctttttggaaa-3**'
	**Antisense: 5'-cgcgtttccaaaaagggccttcatgctatttaatctcgagtttaaatagcatgaaggcccggg-3**'

To create the lentiviral vector encoding firefly luciferase (pLV/PGK-FLuc), the firefly luciferase gene was PCR-amplified from the commercially available pGL3-Control vector (Promega, Madison, WI, USA) using primers 5'-aaaggatccatggaagacgccaaaaaca-3' and 5'-aaactcgagttacacggcgatctttccgc-3'. The PCR product was cloned into the lentiviral vector, pCCL.WPS.PGK-Puro.WHV (described in [33]), using the BamHI/XhoI sites, replacing the puromycin resistance gene. To revert the 5-bp modification in the H1 promoter in the pCCL-PGK-Puro-H1-MCS-based shRNA-expressing vectors to the sequence of the original vector (pCCL-cPPT-PGK-puro-WPRE-LTR-H1-shTNFα, harboring the remains of a BglII site), an overlap extension PCR between two fragments flanking the 5-bp region was done. For both shRNA-encoding lentiviral vectors, the 5' fragment was amplified by primers 5'-ctgctgccggctctgcggcc-3' and 5'-ggggatctgtggtctcatacagaactta-3'. The 3' fragment was amplified using primers 5'-gttctgtatgagaccacagatccccgcagacagatgc-3' and 5'-ctctagcagcatctagagcg-3' (in case of pCCL-PGK-Puro-H1-shIL12B #5) and 5'-gttctgtatgagaccacagatccccgatgggaacgca-3' and 5'-ctctagcagcatctagagcg-3' (in case of pCCL-PGK-Puro-H1-shIL12B #6). Overlap extension PCRs between the 5' fragment and the 3' fragments were done by primers 5'-ctgctgccggctctgcggcc-3' and 5'-ctctagcagcatctagagcg-3'. The overlap extension PCR amplicons were inserted into the original lentiviral vectors using the XbaI and ApaI restriction sites. All constructs were verified by sequencing with BigDye^® ^Terminator v.3.1 Cycle Sequencing Kit (Applied Biosystems, Foster City, CA, USA).

### shRNA oligonucleotide design

The lentiviral construct encoding the irrelevant shRNA was constructed as described in [8]. For shIL-12B #1-#7, the loop region in the shRNAs were chosen on the basis of that proposed by McIntyre and Fanning [34]. The design of the shRNA oligonucleotides was to resemble the endogenous situation of the H1 promoter and the H1 RNA coding region (the RNA Component of Human RNase P). This includes a distance of 26-nt between the TATA-box and the transcriptional start site, three cytosines before the transcriptional start and five consecutive thymines functioning as a termination signal [35]. The cleavage of the shRNA transcript at the termination site is after the second uridine yielding a 2-nt 3' overhang resembling that of endogenously processed pre-miRNAs [29]. shIL12B #1 was adapted from an siRNA reported by Flynn *et al. *to target a sequence of the murine IL-12B with high homology to the human sequence [36]. The target sequence was modified to match the human sequence. shIL12B #2 was adapted from an siRNA that has been reported by Borg *et al. *to efficiently target human IL-12B [37]. shIL12B #3-#7 were predicted by online algorithms: shIL12B #3-#5 by Dharmacon's siDESIGN, shIL12B #6 by InvivoGen siRNA Wizard, and shIL12B #7 by Qiagen's BioPredSi. See table 1 for shRNA oligonucleotide sequences.

### Cell culturing

HeLa, HEK293, and 293T cells were cultured at standard conditions at 37˚C in 5% (v/v) CO_2 _and maintained in Dulbecco's modified Eagle's medium (Cambrex, Verviers, Belgium) supplemented with 10% fetal calf serum, penicillin (100 U/ml), streptomycin (0.1 mg/ml), and L-glutamine (265 mg/l).

### Dual-Luciferase Reporter Assay

For co-transfection experiments, HEK293 cells were seeded in 24-well plates (1.9 × 10^4 ^cells/well) one day before transfection. Co-transfections were performed with a total of 0.4 μg DNA (0.36 μg shRNA-encoding lentiviral vector and 0.04 μg psiCHECK-IL12B vector) using FuGENE-6 (Roche, Basel, Switzerland) according to the manufacturer's protocol. Forty-eight hours post-transfection, Renilla and Firefly Luciferase activities were analyzed by the use of the Dual-Luciferase^® ^Reporter Assay System (Promega, Madison, WI, USA) according to the manufacturer's protocol. Reactions were carried out in 96-well plates and luminescence readings were performed in a multisample platereading luminometer (Berthold, Bad Wild-bad, Germany). Renilla Luciferase activity was normalized to Firefly Luciferase activity and presented relative to the negative vehicle control (pCCL-PGK-Puro-H1-MCS).

In transduction studies of shRNA-encoding lentiviral vectors, HEK293 cells were seeded in 24-well plates (1.9 × 10^4 ^cells/well) and transduced at an MOI of 10. The viral supernatant was supplemented with 8 μg/ml polybrene (Sigma-Aldrich, Milwaukee, WI). One day post-transduction, transfections with 0.04 μg psiCHECK-IL12B vector using FuGENE-6 (Roche, Basel, Switzerland) were performed according to the manufacturer's protocol and luciferase activities were measured forty-eight hours post-transfection as described above. To make cell lines with stable expression of shRNAs from a single lentiviral insertion, HEK293 cells were seeded in 6-well plates (5 × 10^4 ^cells/well) one day before transduction. Lentiviral supernatants were serially diluted and supplemented with 8 μg/ml polybrene (Sigma-Aldrich, Milwaukee, WI). Transduced cells were grown in 1 μg/ml puromycin-containing medium (Sigma-Aldrich, Milwaukee, WI) after which cells from a well with a low colony-count were pooled and allowed to expand. Ten days post-transduction, these cells were seeded in 24-well plates (1.9 × 10^4 ^cells/well) and transfected the following day with 0.04 μg psiCHECK-IL12B vector using FuGENE-6 (Roche, Basel, Switzerland) according to the manufacturer's protocol and luciferase activities were measured forty-eight hours post-transfection as described above. All dual luciferase assay experiments were performed at least in triplicates.

### Generation of the IL12B-expressing HeLa cell line, HeLa-IL12B

The Sleeping Beauty DNA transposon vector encoding IL-12B was created by amplification of IL-12B cDNA from the psiCHECK-IL12B vector with primers 5'-aaagcggccgcccattggactctccgtcctg-3' and 5'-aaagcggccgcgattacaaagaagagttttt-3'. eGFP was excised using NotI from a biscitronic DNA transposon vector, pT2/CMV-eGFP(s).SV40-neo. The IL-12B PCR amplicon was inserted into the vector in the sense orientation using the NotI sites. Transposition in the HeLa cell line was performed in 6-well plates into which 2 × 10^5 ^HeLa cells were seeded. Twenty-four hours later, the cells were co-transfected with 0.5 μg transposon vector and 0.5 μg of transposase-encoding vector (SB100X) using FuGene6 (Roche, Basel, Switzerland) according to manufacturer's protocol. The cells were selected for positive transposition by G418-supplemented medium (600 μg/mL) for ten days. From a well with a low colony-count, single clones were isolated to separate dishes and expanded. IL-12B mRNA expression analysis was performed by purification of RNA using the RNeasy Mini Kit (Qiagen, Valencia, California, USA) followed by first strand cDNA synthesis using AffinityScript™ QPCR cDNA Synthesis Kit (Stratagene, La Jolla, California, USA) according to manufacturer's protocol. IL-12B mRNA expression was confirmed by IL12B-specific PCR on first strand cDNA. A single HeLa-IL12B clone was chosen for subsequent experiments.

### Lentiviral production

Lentiviral vectors were produced in 293T cells which were seeded in 10-cm dishes (4 × 10^6 ^cells/well). Twenty-four hours after, cells were calcium phosphate transfected with 3.75 μg pMD.2G, 3 μg pRSV-Rev, 13 μg pMDGP-Lg/RRE, and 13 μg lentiviral transfer vector. Forty-eight hours post-transfection, the viral supernatant was harvested and filtered in 0.45 μm filters to remove cellular debris (Sarstedt, Nümbrecht, Germany). The resulting lentiviral vectors were designated LV-shRNA (e.g. LV-shIL12B #6). Colony-forming titer assays were performed on HEK293 cells seeded in 6-well plates (5 × 10^4 ^cells/well) one day before transduction. Lentiviral supernatants were serially diluted and supplemented with 8 μg/ml polybrene (Sigma-Aldrich, Milwaukee, WI). Transduced cells were grown in 1 μg/ml puromycin-containing medium (Sigma-Aldrich, Milwaukee, WI) for seven days after which the number of colonies were counted in the wells. For *in vivo *transductions of xenografted human skin, the lentiviral vectors were ultra-centrifuged for 2 hours (4°C at 25000 r.p.m.) in a SW28 rotor (Beckman Coulter, Fullerton, CA, USA). Virus pellets were resuspended overnight in PBS (without CaCl_2 _and MgCl_2_) at 4°C in a volume of 1/300 of the original volume. The lentiviral vector yield was determined by measurements of p24 Gag protein using a HIV-1 p24 Antigen ELISA Kit (ZeptoMetrix, Buffalo, NY) according to manufacturer's protocol.

### In vivo treatment of xenografted human skin by shIL12B-encoding lentiviral vectors and by p40-targeting antibodies

For evaluation of luciferase expression in human skin transduced with luciferase-encoding lentiviral vectors, skin grafts from a patient undergoing plastic surgery were obtained. For the evaluation of the effect of IL-12B knockdown in human psoriatic skin by shIL12B #6 or Ustekinumab (Stelera^®^, Janssen-Cilag, Birkerød, Denmark), psoriatic skin biopsies were obtained from seven patients with moderate to severe plaque psoriasis. The psoriasis of the participants was untreated for at least one month prior to biopsy acquisition. Informed patient consent was obtained and the study was approved by the Central Ethical Committee and conducted according to the Declaration of Helsinki protocols. Animal studies were carried out with permission from the Danish Experimental Animal Inspectorate. The xenotransplantation procedure was as follows: each skin biopsy, containing both epidermis and dermis, was split into several grafts (each 1.5 × 1.5 × 0.05 cm) and transplanted onto C.B-17 severe combined immunodeficient (SCID) mice, 6-8 weeks old (Taconic M & B, Silkeborg, Denmark), as described in [7]. Shortly, the mice were anesthetized prior to surgery by a subcutaneous injection of Ketaminol (ketamine, 100 mg/kg; Intervet, Skovlunde, Denmark) and Narcoxyl (xylazine, 10 mg/kg; Intervet, Skovlunde, Denmark). The back was shaved and part of the exposed skin removed. The grafts were sutured with absorbable 6-0 suture (Caprosyn, Covidien, Dublin, Ireland) and covered with Xeroform dressings (Sherwood Medical Company; Markham, Ontario, Canada) for one week. The mice were kept under pathogen-free conditions throughout the study. The grafts healed for 10 days before treatment initiation. To evaluate luciferase expression in skin grafts and the effect of IL-12B knockdown by shRNA delivery, 150 μl LV-PGK-Fluc, LV-shIL12B #6 or LV-shIrrelevant were administered intradermally into the grafts as a single treatment (LV-PGK-Fluc was injected at a dose of 6.45 μg p24 Gag in 150 μL and the shRNA-encoding lentiviral vectors were injected at a dose of 30.1 ± 23.4 μg p24 Gag in 150 μL). As a positive control in the IL-12B knockdown study, Betnovat^® ^(1 mg/g cream, GlaxoSmithKline Pharma) was applied once daily for the duration of the three-week treatment. As an additional negative control, a group of mice was left untreated. To evaluate the effect of p40 inhibition by treating the mice with the p40-targeting antibody, Ustekinumab, 200 μL Ustekinumab (0.9 mg/kg) or 200 μL negative control (0.9%sodium chloride solution) were administered to the mice intraperitoneally once weekly for the duration of the three-week treatment.

### Evaluation of bioluminescence

Three mice receiving lentiviral vectors encoding firefly luciferase and one mouse left untreated were analyzed on day 3, 7, 11, 15, 18, 22, 37, 53, 80, and 98 for bioluminescence. Briefly, the mice were injected intraperitoneally with 200 μL luciferin (15 mg/mL) (Synchem OHG, Felsberg/Altenburg, Germany) and subsequently anesthetized with 3.75% isoflurane (Forene^®^, Abbott Scandinavia AB, Solna, Sweden). Anesthesia was maintained at 2% isoflurane while bioluminescence was analyzed using an IVIS^® ^Spectrum (Caliper Life Sciences, Hopkinton, MA, USA) with the software Living Image^® ^4.0. Ten images were acquired at an interval of 2 minutes and peak-intensity images were selected for subsequent analysis. For quantification of bioluminescence from each skin graft, a region of interest was set with a lower threshold of 25% of maximum luminescence and average luminescence was measured within this region.

### Xenograft evaluation

The semi-quantitative clinical psoriasis scores were assessed for each psoriatic xenograft twice weekly in a blinded fashion and scored according to the average of the following clinical signs: scaliness, induration and erythema. The parameters were scored using a four-point scale: 0, complete lack of cutaneous involvement; 1, slight involvement; 2, moderate involvement; 3, severe involvement. On this scale from 0 to 3, a maximal score of 3 represents severe scale, induration and erythema of the xenografted psoriatic skin. After treatment, biopsies from the centre of the graft were obtained and paraffin-embedded. Employing standard methods, sections were stained histochemically with haematoxylin and eosin. Epidermal thickness was measured at least ten random places from the stratum corneum to the deepest part of the rete pegs on three equally distant cut sections and the average value was calculated.

### Quantitative RT-PCR

Skin biopsies from *in vivo*-transduced xenografted psoriatic skin were snap-frozen in liquid nitrogen and stored at -80°C. Biopsies were transferred to RNAlater-ICE (Ambion, Austin, TX, USA) and stored at -20°C for twenty-four hours prior to RNA isolation. From both *in vitro *cultured cells and skin biopsies, total RNA was extracted using the SV Total RNA Isolation System (Promega, Madison, WI, USA) according to manufacturer's protocol. The skin biopsies were homogenized in the lysis buffer for 2 × 2 minutes at 25 Hz using a TissueLyser (Qiagen, Valencia, California, USA). Isolated RNA was dissolved in nuclease-free water and stored at -80°C. First strand cDNA synthesis was performed using the iScript cDNA Synthesis Kit (Bio-Rad, Hercules, California, USA) according to manufacturer's protocol. Lentiviral transcripts were detected using a PCR amplifying part of the puromycin resistance gene and the woodchuck hepatitis element using primers 5'-caccgtcaccgccgacgtcg-3' (s) and 5'- gtcccggaaaggagctgac-3' (as). IL-12B mRNA levels were assessed by qRT-PCR employing the TaqMan^® ^Gene Expression Master Mix (Applied Biosystems, Foster City, CA) according to manufacturer's protocol. IL-12B mRNA expression was determined using IL-12B primers and probes (FAM-labeled MGB-probes) (Hs01011518_m1, Applied Biosystems, Foster City, CA). IL-12B mRNA levels were normalized to the expression of the reference gene ribosomal protein, large, P0 (RPLP0) using RPLP0-specific primers (FAM-labeled MGB-probes) (Hs99999902_ml, Applied Biosystems, Foster City, CA). Expression of each gene was analyzed in at least duplicates using a LightCycler 480 (Roche, Basel, Switzerland). PCR conditions were 10 minutes at 95°C, 45 cycles of 15 seconds at 95°C and 60 seconds at 60°C. For all qPCR experiments, relative mRNA levels were determined using the relative standard curve method. Briefly, a standard curve for each gene was made from serial dilutions of the cDNA. The standard curve was then used to calculate relative amounts of target mRNA in the samples. Mean mRNA values were calculated and the data summarized as mean + SEM.

### Immunohistochemical stainings

Tissue sections were immuno-stained with monoclonal rabbit anti-human antibodies against CD4 (clone SP 35, Cell Marque, Rocklin, CA, USA) and Ki-67 (Clone SP6, Spring Bioscience, Pleasanton, CA, USA), monoclonal mouse anti-human CD8 (M7103, Dako, Glostrup, Denmark), polyclonal goat anti-human antibodies against Elafin/SKALP (an epithelial proteinase inhibitor) (HP9025, Hycult Biotechnology, Uden, the Netherlands) and human β-defensin (hBD)-2 (an antimicrobial peptide) (500-P161G, Peprotech, London, UK). Elafin/SKALP and hBD-2 are both present in psoriasis skin but not in normal or atopic dermatitis skin [38]. Prior to staining, antigens were retrieved in Tris-EGTA (TEG) buffer (pH = 9). Ki-67 was visualized by ultraview Universal HRP/DAB Detection Kit (Ventana Medical Systems, Tucson, AZ, USA), CD4 and CD8 were visualized by EnVision + System-HRP (DAB) kits for mouse and rabbit primary antibodies (Dako, Glostrup, Denmark). Signal was enhanced by 0.5% copper sulfate and nuclear staining was performed by Mayer's Haematoxylin. Elafin/SKALP was visualized by staining with rabbit anti-goat IgG secondary antibody (A21222, Alexa Fluor^®^, Molecular Probes, Eugene, OR) preceded by blocking of unspecific binding by normal rabbit serum (X0902, Dako, Glostrup, Denmark) and signal enhancing by Image-iT™ FX Signal Enhancer (Molecular Probes, Eugene, OR). hBD-2 was visualized by staining with Vectastain^® ^ABC-AP Kit (AK-5005, Vector Laboratories, Burlingame, CA) and Vector^® ^Red - Alkaline Phosphatase Substrate Kit I (SK-5100, Vector Laboratories, Burlingame, CA), following the manufacturer's instructions. Nuclear staining was performed by mounting samples in Prolong Gold anti-fade reagent (Molecular Probes, Eugene, OR).

### Statistical analyses

The nonparametric Mann-Whitney test was used to test for differences between treatment groups in semi-quantitative clinical psoriasis scores. All other p-values were calculated by a two-tailed Student's t-test to test the null hypothesis of no difference between two compared groups. The assumption of equal variances was tested by the F-test. In all statistical analyses, p-values < 0.05 were considered significant.

## Results

### Amelioration of the psoriatic phenotype in xenografted psoriatic skin following systemic injection of p40-targeting antibodies

**Figure 1:**
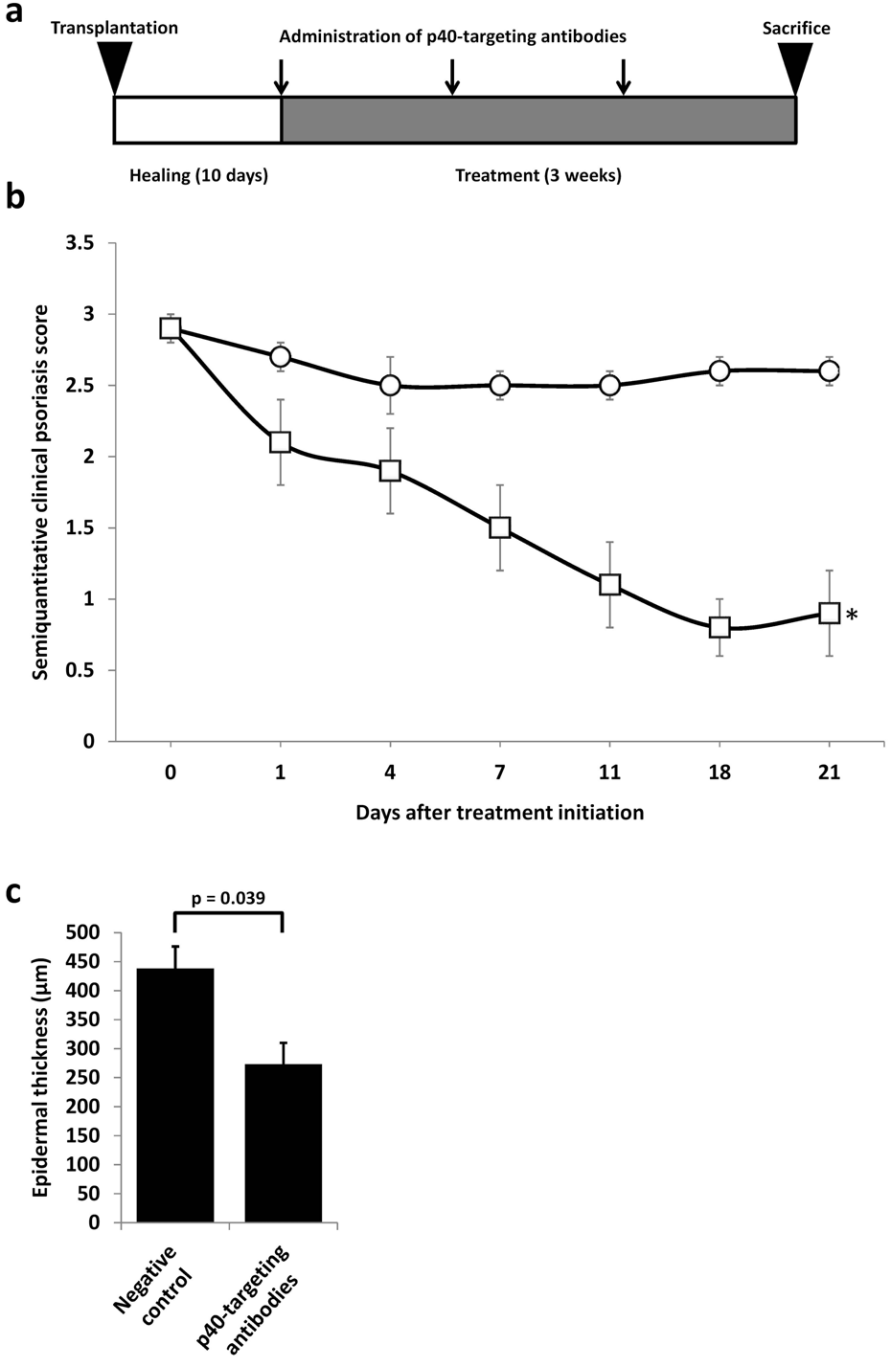
**Amelioration of psoriasis by treatment of mice xenotransplanted with psoriatic skin with systemically administered p40-targeting antibodies**. **(a) **Schematic schedule of treatment with p40-targeting antibodies (Ustekinumab). Psoriatic skin grafts were xenotransplanted onto the back of SCID mice and allowed to heal for ten days. The mice were then treated by weekly intraperitoneal injection of p40-targeting antibodies or negative control. The mice were sacrificed after three weeks of treatment. **(b) **Semiquantitative clinical psoriasis scores were given twice weekly for the three-week treatment duration to mice treated with the negative control (open circles, n = 11) or p40-targeting antibodies (open squares, n = 4). Injections were performed at day 0, 7, and 14. All data points are presented as mean ± SEM. *p = 0.003. **(c) **At treatment endpoint, mice were sacrificed and biopsies from the skin grafts were fixed, paraffin-embedded, H&E-stained, and epidermal thickness was measured in each graft. All data points are presented as mean + SEM.

To evaluate the effects of targeting IL-12B mRNA in psoriatic skin by RNA interference, we wished to employ the psoriasis xenograft transplantation model in which psoriatic skin is grafted onto the back of SCID mice. However, to validate the suitability of this model for studying the therapeutic efficacy of targeting IL-12B mRNA, we first treated mice xenotransplanted with psoriatic skin with the clinically approved p40-targeting antibody, Ustekinumab. Mice were administered either p40-targeting antibodies (n = 4) or negative control (n = 11) (Figure 1a).

The xenografted psoriatic skin was assessed throughout the study and a semiquantitative clinical psoriasis score based on erythema, thickness and scaliness of the psoriatic plaques was recorded twice weekly in a blinded fashion. After three weeks the mice were sacrificed and endpoint evaluation of the treatment of psoriasis was determined histologically by measuring epidermal thickness on three equally distantly cut hematoxylin and eosin-stained sections of the graft. All sections were blinded prior to measurements and evaluated randomly. As evident from Figure 1b, treatment with p40-targeting antibodies led to a significant improvement of the clinical phenotype of the psoriatic skin compared to the negative control. This was confirmed by measurements of the epidermal thickness of the skin grafts at treatment endpoint (Figure 1c). Average epidermal thickness of the skin grafts were reduced by 38% (438 mm to 273 mm) in the group treated with p40-targeting antibodies compared to the negative control group (p = 0.039). In conclusion, the high therapeutic potency of p40-targeting antibodies validates the relevance of studying p40-targeting in the xenograft transplantation model.

### Potency screening of a panel of IL12B-targeting shRNAs

**Figure 2:**
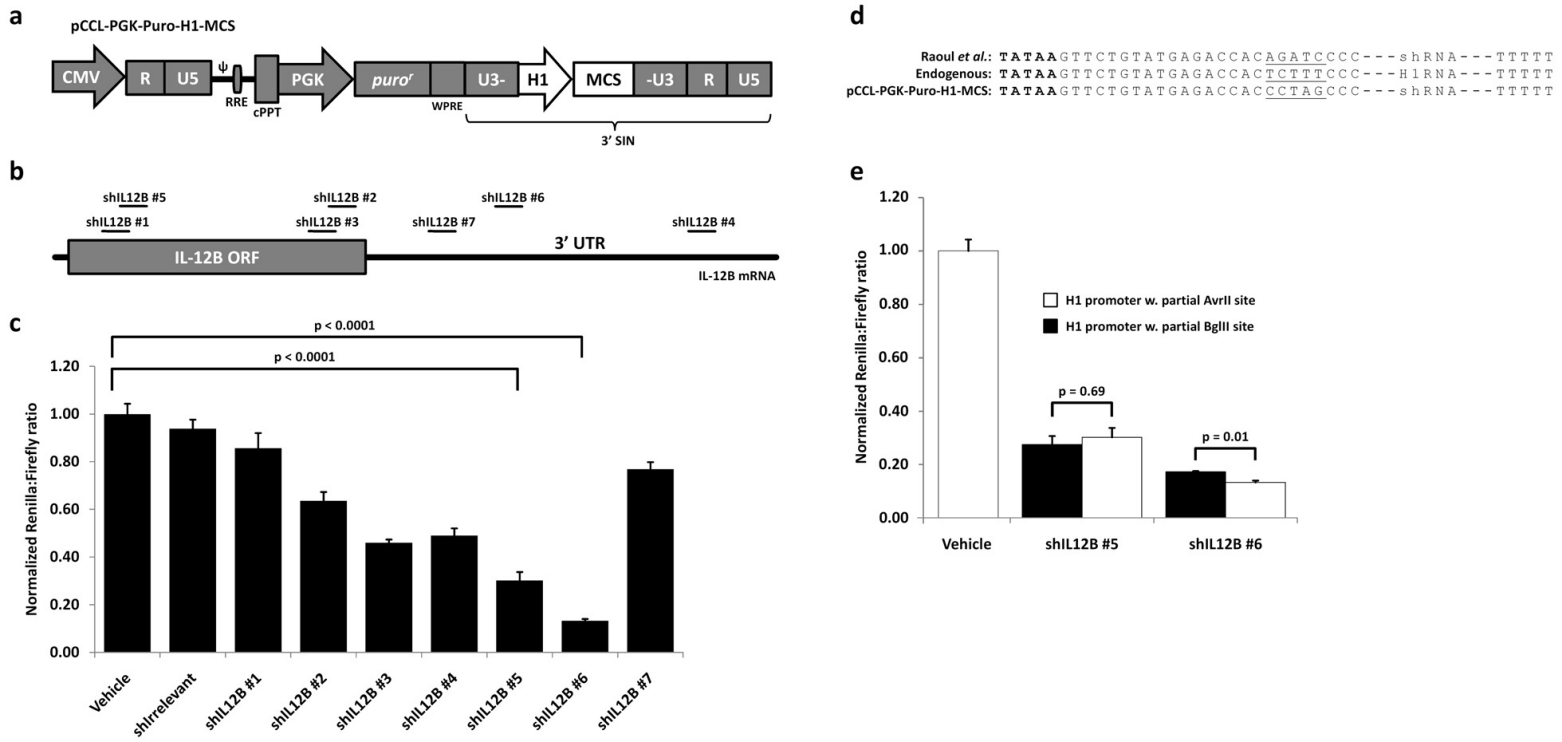
**Development of a lentiviral vector for an easy one-step shRNA oligonucleotide cloning procedure and potency screening of a panel of shRNAs targeting human IL-12B**. **(a) **Schematic overview of the lentiviral vector, pCCL-PGK-Puro-H1-MCS, for an easy one-step shRNA cloning procedure. shRNA oligonucleotides with compatible overhangs can be cloned into the multiple cloning site (MCS) from which shRNA expression will be driven by the H1 promoter. **(b) **Schematic overview of IL-12B mRNA and the target sites for the seven designed shRNAs. **(c) **Potency screening of seven shRNAs targeting IL-12B mRNA using the dual luciferase assay. HEK293 cells were co-transfected with the shRNA-encoding lentiviral vector and the psiCHECK-IL12B vector encoding firefly luciferase for transfection normalization and a fusion mRNA consisting of renilla luciferase and IL-12B. Luciferase activities were measured forty-eight hours post-transfection and renilla luciferase activity was normalized to firefly luciferase activity and depicted relative to transfection with the empty lentiviral vector, pCCL-PGK-Puro-H1-MCS, not encoding an shRNA (vehicle). **(d) **Sequence comparison of the 3' end of the H1 promoter depicting sequence differences introduced in the H1 promoter for cloning of shRNA oligonucleotides. The TATA box is depicted in bold, and sequence differences are underlined. Furthermore, the termination signal of the H1 promoter consists of 5 thymines. **(e) **Confirmation of full promoter activity from the H1 promoter of pCCL-PGK-Puro-H1-MCS modified to encompass a multiple cloning site for easy one-step cloning of shRNA oligonucleotides. shRNA potency was evaluated when expressed from the two sequence contexts of the H1 promoter, namely the context described by Raoul et. al. (black bars) and the new sequence context of the pCCL-PGK-Puro-H1-MCS (white bars). Comparison of shRNA potency was performed using the dual luciferase assay as described above. All dual luciferase assay experiments were performed at least in triplicates and data are depicted as mean + SEM.

For cytokine knockdown by RNAi, we have previously utilized a lentiviral vector, originally described by Raoul *et al. *[32], in which shRNA expression is driven by the H1 promoter situated in the 3' LTR of the lentiviral vector. This design allows duplication of the shRNA expression cassette upon reverse transcription of vector RNA [8]. However, shRNA oligonucleotide cloning into this vector was an elaborate process involving three cloning steps. To ease this process we developed a vector (pCCL-PGK-Puro-H1-MCS) with a multiple cloning site immediately downstream of the H1 promoter into which annealed shRNA oligonucleotides with compatible overhangs could be directly cloned (Figure 2a). The generation of the pCCL-PGK-Puro-H1-MCS vector improved the shRNA oligonucleotide cloning procedure significantly, supporting a faster and easier functional screening of lentivirally delivered shRNAs.

To identify potent shRNA variants targeting IL-12B mRNA, seven target sites in the IL-12B mRNA sequence were chosen, either predicted by online algorithms or adapted from previously published efficacious siRNAs. Four target sequences were located in the coding sequence and three were located in the 3' UTR (Figure 2b). To monitor shRNA knockdown potencies, each of the seven shRNA sequences were cloned into pCCL-PGK-Puro-H1-MCS and analyzed for the ability to target the IL-12B sequence by using a dual luciferase expression assay. By this approach, expression of the renilla luciferase reporter gene, when fused to the IL-12B sequence, was responsive to anti-IL12B shRNAs, whereas expression of the firefly luciferase from the same plasmid allowed normalization for transfection variations.

HEK293 cells were co-transfected with the psiCHECK-IL12B plasmid and the plasmid encoding the lentiviral vector used as an expression plasmid for episomal shRNA expression. Luciferase activities were measured forty-eight hours post-transfection and shRNA potencies were determined by normalizing to the level of expression obtained with the lentiviral plasmid not expressing any shRNA, utilized here as a negative control (vehicle). An shRNA not matching any known sequence in the human genome was used as an additional negative control (shIrrelevant) [39]. As seen in Figure 2c shRNA potencies varied greatly, ranging from almost inactive to 87% down-regulation of the fusion transcript as seen for shIL12B #6.

### Verification of full H1 promoter activity in the pCCL-PGK-Puro-H1-MCS vector

In the making of the lentiviral vector, pCCL-PGK-Puro-H1-MCS, for an easy one-step cloning of shRNA oligonucleotides, five base-pairs immediately upstream of the transcriptional initiation site in the H1 promoter were modified to accommodate the MCS, leaving the remains of an AvrII restriction site instead of that of a BglII restriction site (Figure 2d). It should be noted that the original lentiviral vector already harbored a 5-bp modification compared to the endogenous wild-type H1 promoter, indicating that this 5-bp region did not contain any *cis*-acting elements required for H1 promoter activity. However, to fully verify that shRNA expression and potency was not affected by this modification, the lentiviral constructs encoding the two most potent anti-IL12B shRNAs (#5 and #6) were reverted back to the original sequence (BglII) as reported by Raoul *et al. *[32] by PCR site-directed mutagenesis. The two constructs with the remains of an AvrII and a BglII restriction site, respectively, were compared in the dual luciferase assay (Figure 2e). The two constructs encoding shIL12B #5 did not display any difference in shRNA potency. When expressed from the context of the new vector containing the shRNA insertion linker, shIL12B #6 elicited a marginally improved knockdown of the fusion transcript, indicating that the 5-bp mutation did not negatively affect shRNA expression and potency.

### Stable IL-12B down-regulation following lentiviral transduction

To certify that the gene cassette encoding shIL12B #6 was efficiently transferred by the lentiviral vector system, we first measured the transductional efficiency of the shRNA-expressing vector in experiments that included the pCCL-PGK-Puro-H1-MCS vector as a control. High transduction titers of shRNA-expressing lentiviral vectors were confirmed (ranging from 1.6 × 10^6 ^to 5.3 × 10^6 ^colony-forming units per mL [cfu/mL] on HeLa cells), although the titers on average were reduced 4-fold relative to the control vector which did not express an shRNA (data not shown). Such findings are in agreement with previous studies that reported 5- to 6-fold decrease in viral titers presumably caused by shRNA expression and consequent targeting of the viral RNA genome during viral vector production [30].

**Figure 3:**
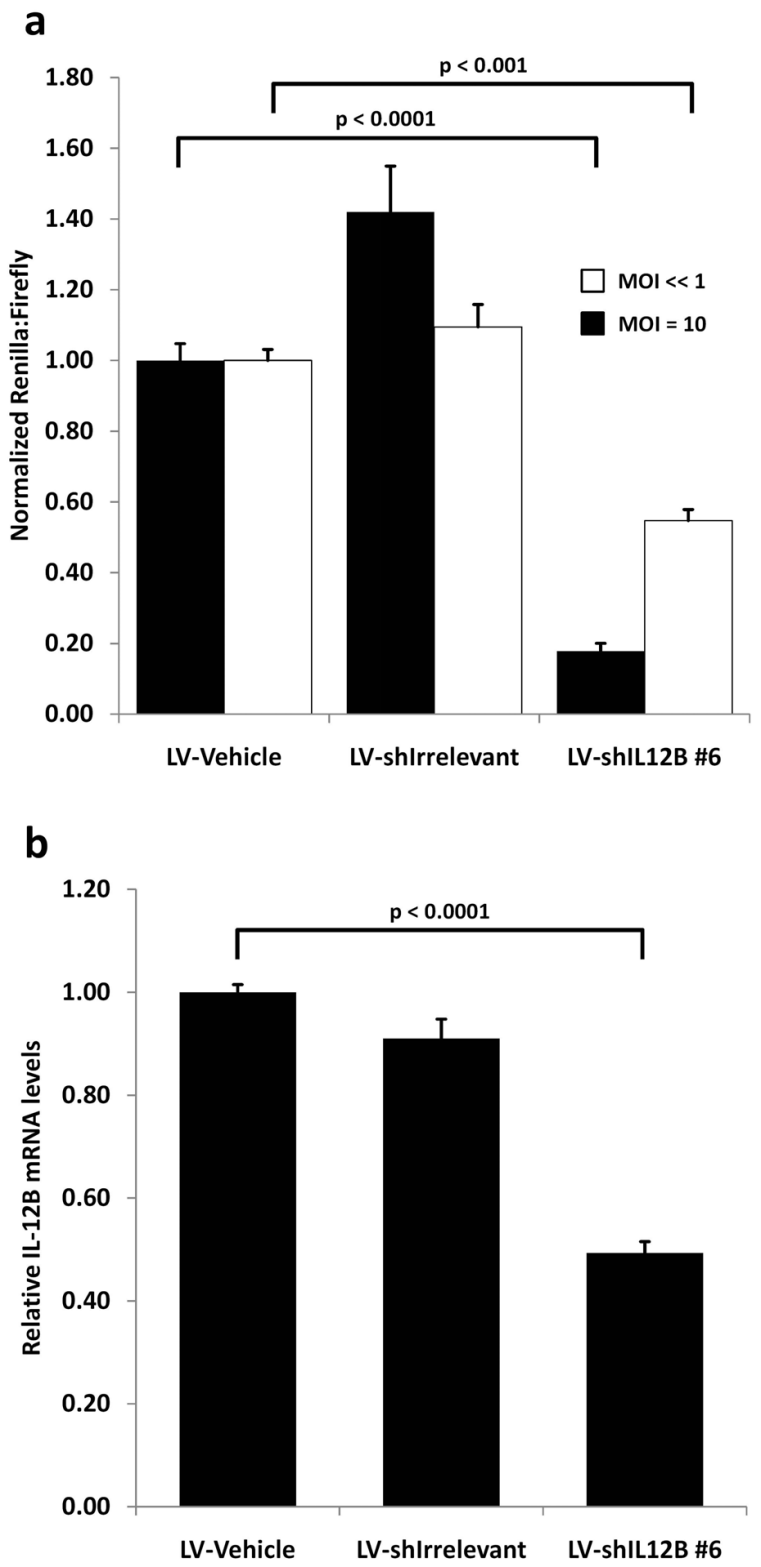
**Confirmation of shRNA potency after lentiviral delivery**. **(a) **shRNA potency evaluation after transduction with shRNA-encoding lentiviral vectors. HEK293 cells were transduced at an MOI of 10 (black bars) or << 1 (white bars) followed by puromycin selection for ten days to ensure that cells harbored only a single lentiviral insertion. In the two cases, cells were transfected with the psiCHECK-IL12B vector one day and ten days post-transduction, respectively, and luciferase activities were measured forty-eight hours post-transfection. Renilla luciferase activity was normalized to firefly luciferase activity and depicted relative to transduction with lentiviral vectors not encoding an shRNA (vehicle). **(b) **An IL-12B expressing HeLa cell line was transduced with shRNA-encoding lentiviral vectors at an MOI of 2. IL-12B mRNA levels were evaluated by qRT-PCR two days post-transduction. All assay were performed at least in triplicates and data are depicted as mean + SEM.

To evaluate shRNA potency when delivered by lentiviral vectors, HEK293 cells were transduced at a multiplicity of infection (MOI) of 10 (IU/cell, infectious units per cell) or at an MOI of << 1 (followed by a ten-day puromycin selection) to ensure that cells harbored only a single lentiviral insertion. The cells were then transfected with the psiCHECK-IL12B vector one day and ten days post-transduction, respectively, and luciferase activities were evaluated forty-eight hours post-transfection. Knock-down efficiencies of the fusion transcript are shown in Figure 3a, and it is evident that the potency of shIL12B #6 was maintained when delivered by lentiviral transductions at an MOI of 10. shIL12B #6 mediated a knockdown of 82% which is comparable to the 87% observed when the shRNAs were expressed episomally from the transfected plasmid. shRNA expression from a single lentiviral insertion mediated a knockdown of 45% of the fusion transcript, clearly demonstrating the dose-dependency of shRNAs in mediating RNAi.

To evaluate knockdown of an endogenously expressed target, which was not in the context of a fusion mRNA, an IL-12B expression cassette was inserted into the HeLa cell line by the means of the Sleeping Beauty DNA transposon system [40]. IL-12B mRNA expression from the genomically inserted DNA transposon vector was confirmed by RT-PCR (data not shown), and the cell line was transduced with the shRNA-encoding lentiviral vectors at an MOI of 2. Endogenously expressed IL-12B mRNA was efficiently targeted by the lentivirally delivered shIL12B #6. The knockdown potency was evaluated by quantitative RT-PCR analysis of the transduced cells harvested two days post-transduction. We detected a 51% knockdown of IL-12B mRNA transcripts (Figure 3b) which demonstrated a high potential of lentiviral delivery of IL12B-directed shRNAs for efficient targeting of aberrantly expressed IL-12B for RNAi-mediated down-regulation.

### Efficient and persistent cutaneous transgene expression following intradermal injection of lentiviral vectors in xenografted human skin

**Figure 4:**
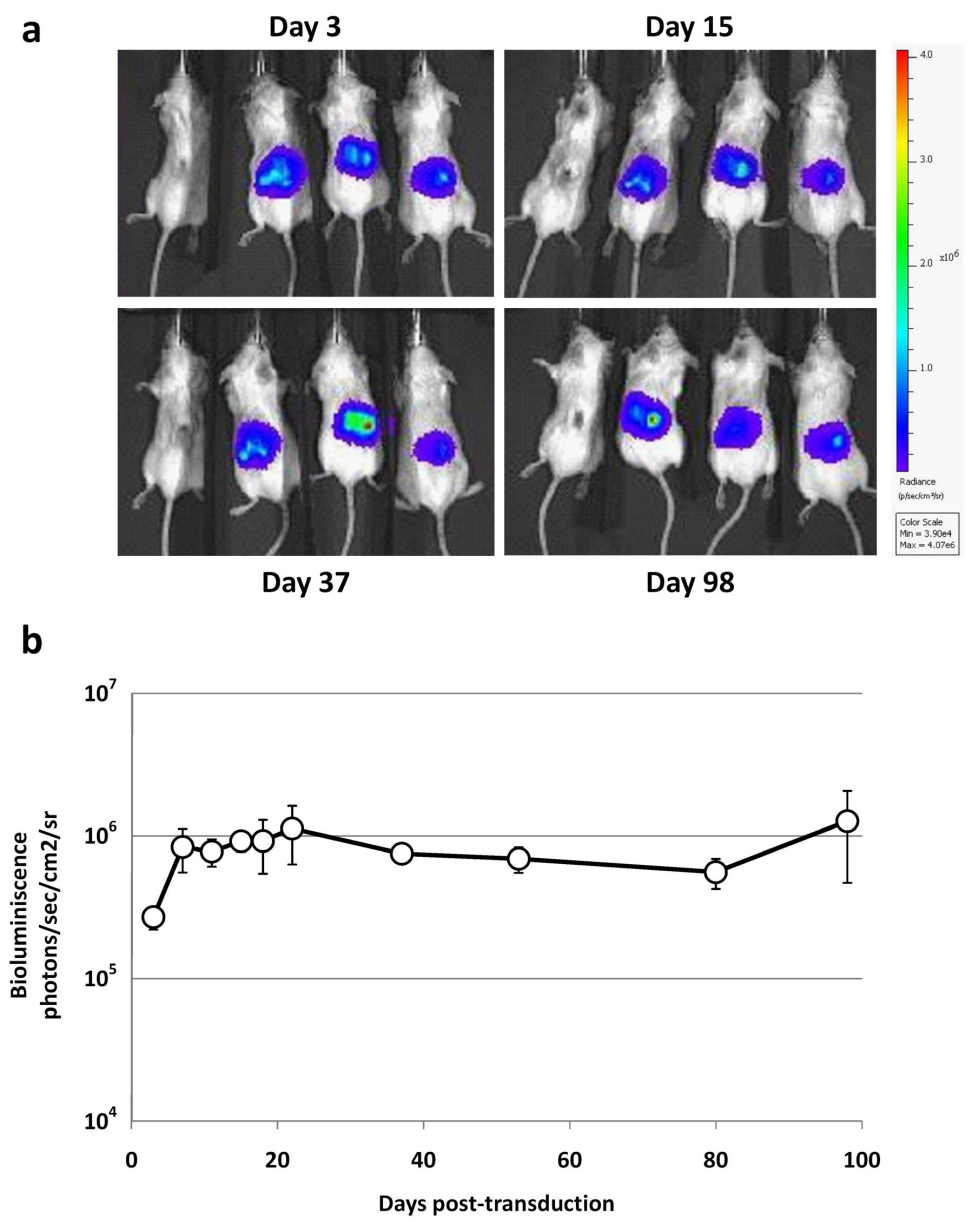
**Efficient and persistent transgene expression after lentiviral transduction of xenografted human skin**. Normal human skin grafts were xenografted onto the back of four SCID mice and three mice were injected a single intradermal dose of firefly luciferase-encoding lentiviral vectors (three mice to the right in each picture). **(a) **Representative images of the four mice showing high bioluminescence at day 3, 15, 37, and 98 from the xenografted human skin transduced with luciferase-encoding lentiviral vectors. **(b) **Bioluminescence from the xenografted human skin on the three mice injected with luciferase-encoding lentiviral vectors was measured at various time points after transduction. Data are depicted as mean ± SEM.

We have previously documented efficient transgene delivery to xenografted psoriatic skin as confirmed by high expression of green fluorescent protein (GFP) in the epidermis and to some extent in the dermis, three days after intradermal injection of eGFP-encoding lentiviral vectors [8]. To evaluate the persistency of transgene expression, luciferase-encoding lentiviral vectors were intradermally injected in normal human skin xenografted onto the back of SCID mice. We detected bioluminescence from the transduced skin grafts for the duration of the experiment (98 days) demonstrating high stability and persistency of reporter gene expression after a single vector injection (Figure 4a and 4b). Also, we did not measure any bioluminescence in the non-transduced grafts or from other tissues of the vector-treated mice (Figure 4a), demonstrating that vector transduction was confined to the skin grafts without apparent spreading of the injected lentiviral vector.

### Targeting of IL-12B mRNA in xenografted psoriatic skin by lentiviral delivery of anti-IL12B shRNAs

**Figure 5:**
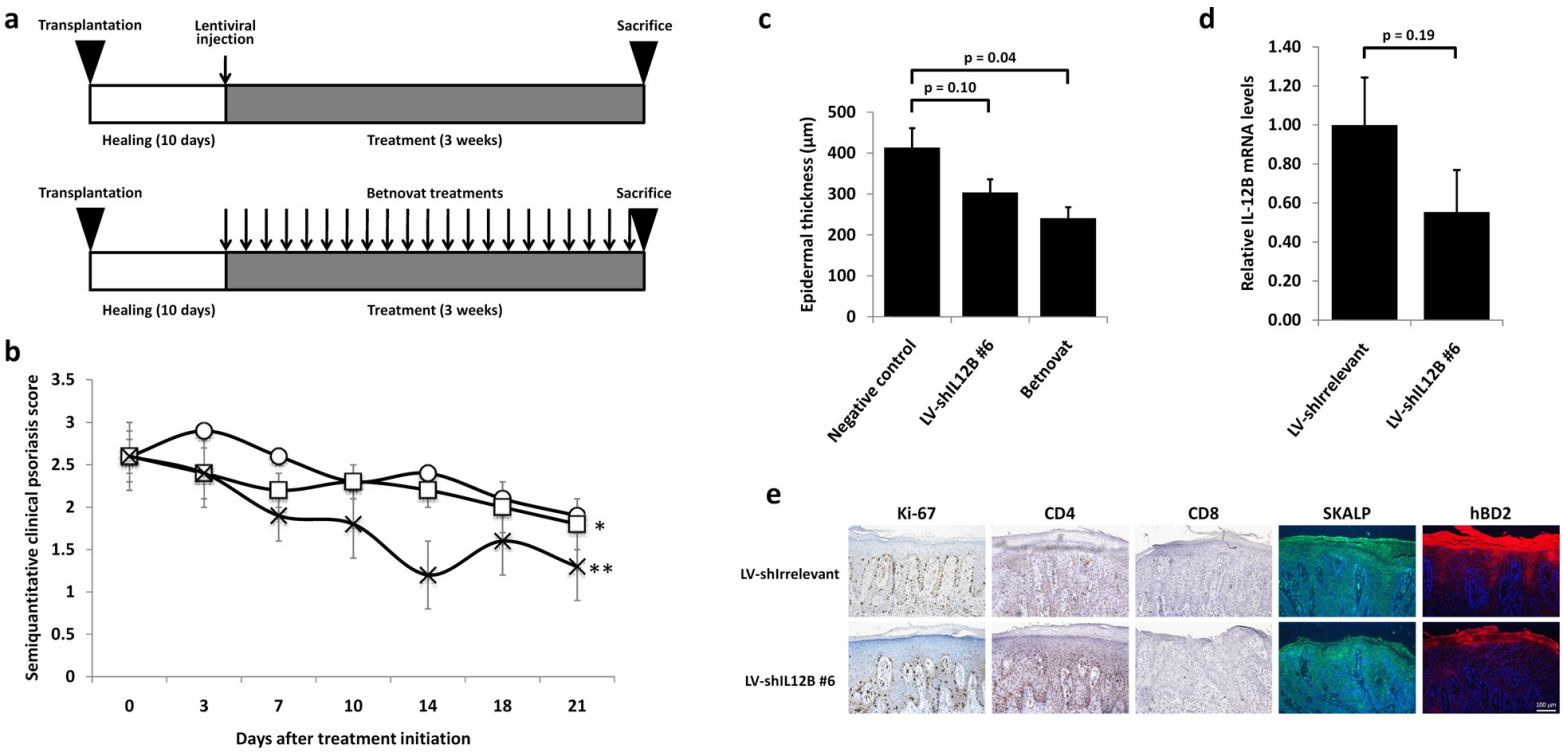
***In vivo *knockdown of IL-12B in xenografted psoriatic skin by lentiviral delivery of anti-IL12B shRNAs**. **(a) **Schematic schedule of treatment with shRNA-encoding lentiviral vectors (upper panel) and topically applied steroid (lower panel). Psoriatic skin grafts were xenografted onto the back of SCID mice and allowed to heal for ten days. The skin grafts were then either left untreated or treated by a single intradermal injection of lentiviral vectors encoding either shIL12B #6 or an irrelevant shRNA, or treated daily with the topically applied class three steroid, Betnovat (positive control). The mice were sacrificed three weeks after treatment. The two groups consisting of untreated mice and mice treated with lentiviral vectors encoding an irrelevant shRNA were pooled to a single group (negative control) due to high similarities in the semiquantitative clinical psoriasis scores and epidermal thicknesses. **(b) **Semiquantitative clinical psoriasis scores were given twice weekly for the three-week treatment duration to mice treated with negative control (open circles, n = 16), LV-shIL12B #6 (open squares, n = 11), or Betnovat (crosses, n = 6). Intradermal injections were performed at day 0. All data points are presented as mean ± SEM. *p = 0.86, **p = 0.12. **(c) **At treatment endpoint three weeks post-transduction of the skin grafts, mice were sacrificed and biopsies from the skin grafts were fixed, paraffin-embedded, H&E-stained, and epidermal thickness was measured in each graft. **(d) **Biopsies from the xenografted psoriatic skin injected with shRNA-encoding lentiviral vectors were acquired at treatment endpoint three weeks post-transduction and evaluated for IL-12B gene expression by qRT-PCR. Data are presented as mean + SEM. **(e) **Immunohistochemical stainings were performed for Ki-67, CD4, CD8, SKALP/Elafin, and hBD2 on skin sections treated with LV-shIrrelevant or LV-shIL12B #6.

To evaluate the effects of targeting IL-12B mRNA in psoriatic skin, mice xenografted with psoriatic skin were divided into 4 groups. The first group (n = 11) was administered a single intradermal dose of lentiviral vectors encoding shIL12B #6 (Figure 5a). The second group (n = 9) was administered a single dose of lentiviral vectors encoding the irrelevant shRNA (shIrrelevant). The third group (n = 7) was left untreated and, finally, the last group of mice (n = 6) was treated daily with the topically applied class three glucocorticoid steroid Betnovat (betamethasone valerate) (Figure 5a), which is a local treatment used in the clinic to treat mild to moderate psoriasis due to its anti-inflammatory and immunosuppressive properties. At treatment endpoint, RNA was purified from the skin grafts and RT-PCR confirmed the presence of lentiviral transcripts in the skin (data not shown), corroborating the previous results, that expression from lentivirus-mediated integration is persistent during the 3-week treatment period.

As in the Ustekinumab study, the xenografted psoriatic skin was assessed throughout the study and assigned a semiquantitative clinical psoriasis score twice weekly in a blinded fashion. The mice were sacrificed after three weeks and treatment endpoint evaluation was determined by measuring epidermal thickness and by quantifying IL-12B mRNA levels in biopsies obtained from the psoriatic skin grafts. Since skin grafts treated with lentiviral vectors encoding shIrrelevant and the untreated skin grafts obtained similar semiquantitative clinical psoriasis scores for the duration of the experiment (p > 0.35 for all time points) and displayed similar epidermal thickness (p = 0.22) as well as similar degree of psorasiform papillae, Monroe's absess, vessel formation and parakeratosis, these two groups were pooled to one group, designated 'Negative control'.

As evident from Figure 5b, treatment with shIL12B-encoding lentiviral vectors did not seem to have an effect on the clinical phenotype of the psoriatic skin compared to the negative control. On day 3 and 7 we observed a difference in semiquantitative clinical psoriasis score of 0.5 and 0.4, respectively (p = 0.074 and p = 0.069) indicating a slight amelioration of the psoriatic phenotype. Treatment with the positive control, Betnovat, improved the psoriatic phenotype during the treatment course, however, the improvement at treatment endpoint was not significant (p = 0.12). Average epidermal thicknesses are shown in Figure 5c which shows that grafts injected with shIL12B-encoding lentiviral vectors exhibited a 27% reduction (414 mm to 304 mm) in average epidermal thickness compared to the negative control group. However, this reduction did not appear to be significant (p = 0.10). Betnovat, as expected, elicited a 42% reduction in epidermal thickness (p = 0.04). In addition to the epidermal thickness, IL-12B mRNA levels were quantified by qRT-PCR in the skin grafts. As evident from Figure 5d, treatment with shIL12B-encoding lentiviral vectors elicited a 45% reduction of IL-12B mRNA in the skin grafts in average. However, as in the case of epidermal thicknesses, this apparent reduction was not supported by statistical analysis (p = 0.19) probably ascribable to the limited number of skin grafts.

Finally, we carried out immunohistochemical stainings for characteristic molecular markers of psoriatic skin (Figure 5e). Neither stainings for Elafin/SKALP and hBD-2, which are both present in psoriatic but not in normal skin, nor for the cell proliferation marker Ki-67 revealed any effect of the treatment with shIL12B #6 relative to the shIrrelevant control. Furthermore, staining of skin-homing T-cells by use of antibodies targeting CD4 and CD8 did not show a difference between skin grafts treated with shIL12B #6 and shIrrelevant.

In conclusion, we saw only a marginal improvement of the psoriatic phenotype within the first week after efficient intradermal administration of lentiviral vectors encoding the highly potent IL12B-targeting shRNA. Measurements of epidermal thickness and IL-12B mRNA levels showed reductions of 27% and 45%, respectively, indicating that the vector-encoded shRNAs were actively targeting IL-12B mRNA in the skin. In summary, our data suggest that intradermally administered IL12B-targeting shRNAs impact the levels of IL-12B mRNAs and result in a reduced epidermal thickness that are very similar to the effects we have previously reported for shRNAs targeting TNFα mRNA [8]. However, the psoriatic phenotype was not significantly affected by targeting IL-12B mRNA in the psoriasis xenograft transplantation model.

## Discussion

Our research group has previously provided evidence of the therapeutic efficacy of *in vivo *knockdown of TNFα mRNA in human skin facilitated by DNA-encoded shRNA molecules [8]. This proof-of-concept study confirmed the therapeutic applicability of shRNA expression in skin and documented the potential use of RNAi in the treatment of psoriasis. In the present study, we extend the exploration of RNAi-mediated cytokine knockdown in human skin to include the common subunit of IL-12 and IL-23, p40, encoded by the IL-12B gene. The p40 subunit is a clinically validated therapeutic target in psoriasis, but it remains unclear if targeting IL-12B mRNA by RNAi-mediated degradation is therapeutically relevant.

The potency of any given shRNA is highly dependent on target sequence and context. Therefore, initial screening of a panel of shRNAs is necessary to identify a highly active variant. Indeed, in the initial luciferase-based screening of seven shRNAs targeting IL-12B, one shRNA candidate was identified, which was able to mediate a solid down-regulation of the renilla luciferase gene fused to full length IL-12B cDNA when expressed from either shRNA-encoding plasmids or lentiviral vectors. High shRNA potency was confirmed when the shRNA was expressed from a single lentiviral integration and after extrachromosomal non-integrated lentiviral vectors had been lost. Additionally, the highly varying potencies of the seven shRNA candidates confirmed the general need to improve the *in silico *algorithms for rational target prediction to minimize the initial screening work load in the identification of potent shRNA candidates. Furthermore, we found that the two already published siRNAs that efficiently target the murine [36] and human IL-12B [37] sequences, respectively, could not be successfully adapted to an shRNA context and maintain potency. We explain this by species sequence and target context variations and also by the fact that the efficiency of shRNA processing might also be sequence-dependent which could adversely affect the potency of an siRNA in an shRNA context.

Variations in target accessibility in the fusion mRNA context could influence RNAi activity and thus provide a false measurement of shRNA potency. This emphasizes the need to validate shRNA potency on native endogenously expressed targets. Moving the experimental setup to endogenously expressed targets showed that shRNA potencies were maintained even when shRNAs were expressed from few lentiviral insertions. High shRNA potency at low cellular concentrations is highly relevant for *in vivo *transduction of a tissue where there are a high number of target cells and cell accessibility is hampered compared to *in vitro *grown cells in monolayer.

The psoriasis xenograft transplantation model currently appears to be the best tool to screen anti-psoriatic therapeutic strategies in psoriasis before introducing them into to the clinic [41]. Many already established anti-psoriatic therapies show similar results in the psoriasis xenotransplantation model as in the clinic. In line with these findings, we validated that the clinically approved p40-targeting antibody, Ustekinumab, led to a significant improvement of the psoriatic phenotype as evaluated by the semi-quantitative clinical psoriasis score and measurements of epidermal thickness in the psoriasis xenograft transplantation model.

Using the psoriasis xenograft transplantation model, we wished to evaluate if lentiviral delivery of shIL12B #6 could lower IL-12B mRNA levels and if this would have a beneficial effect on the disease phenotype. We found that IL-12B mRNA levels were reduced by 45% in the shIL12B-receiving skin grafts compared to the negative control. This result pointed towards the establishment of a stable knockdown of IL-12B which is consistent with our previous *in vivo *study of TNFα knockdown in human skin [8]. Dendritic cells and macrophages in the upper dermal compartment (papillary dermis), and to some extent in the epidermis, have been shown to be the main source of IL-12 and IL-23, but epidermal keratinocytes also express both interleukins to a lesser extent [16]. We have previously established that lentiviral vector-mediated eGFP transfer to xenografted psoriatic skin is more efficient in the epidermal compartment than the dermal compartment [8] and in our current setup, this could mean that interfering with IL-12B production in dermal dendritic cells and macrophages was limited by poor shRNA delivery. In contrast, the efficient transduction of epidermal keratinocytes implied that IL-12B knockdown in these cells was not limited by shRNA delivery.

Epidermal keratinocytes in psoriatic lesions have a transit time of 4-7 days from the basal layer to the stratum corneum meaning that the keratinocytes have been replenished several times during the three-week treatment [42]. Hence, in order to stably knock down aberrant levels of IL-12B produced by epidermal keratinocytes throughout the treatment course, an efficient transduction of epidermal stem cells is required. Indeed, persistence of transgene expression from lentivirally transduced xenografted normal human skin was shown beyond the time span of keratinocyte transition from the basal membrane to the stratum corneum, implying that skin stem cells in the basal compartment were efficiently transduced (Figure 4).

Increased epidermal thickness is an important diagnostic hallmark of psoriasis and stable IL-12B knockdown seemed to result in a reduction in epidermal thickness by 27% (414 mm to 304 mm) in skin transduced with shIL12B-encoding lentiviral vectors compared to controls. This finding could be indicative of altered epidermal cell kinetics although no difference was seen in the immunohistochemical staining for the proliferative marker Ki-67. Nevertheless, a resulting clinical disease improvement was not apparent at treatment end-point even though a strong tendency towards this was seen at day 3 and 7 in the treatment course.

**Figure 6:**
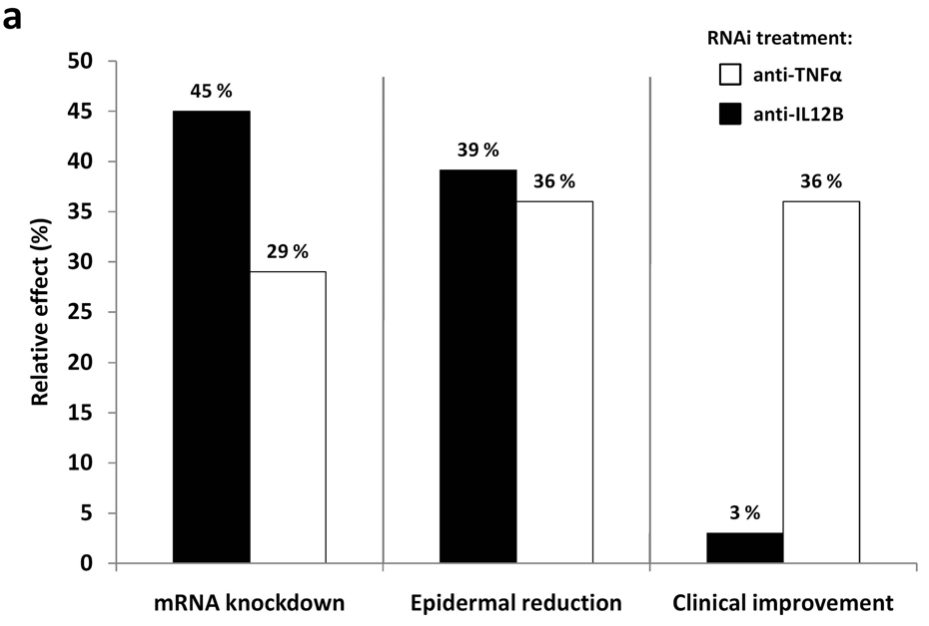
**Comparison of *in vivo *knockdown of IL-12B and TNFα mRNA in xenografted psoriatic skin by lentiviral delivery of anti-IL12B and anti-TNFα shRNAs**. Comparison of the effect of targeting IL-12B mRNA (black bars) and TNFα mRNA (white bars) in xenografted psoriatic skin by lentiviral delivery of cytokine-targeting shRNAs. mRNA knockdown: bars depict percentage down-regulation of IL-12B and TNFα mRNA, respectively, in the transduced grafts compared to the negative control group. Epidermal thickness: bars depict percentage reduction of epidermal thickness compared to the negative control (100% indicates reduction of epidermal thickness to that of average non-lesional skin). Clinical improvement: bars depict improvement of the semiquantitative clinical psoriasis score at treatment endpoint compared to the negative control (100% indicates complete disease resolution to non-lesional skin).

We have previously shown that TNFα mRNA knockdown in the psoriasis xenograft transplantation model has a significant positive effect on the clinical psoriatic phenotype throughout the treatment course [8]. When compared to the present study, knockdown of target mRNAs (IL-12B and TNFα mRNA, respectively) and the following reduction in epidermal thickness were comparable (Figure 6) [8]. This indicates that both cytokines can be efficiently targeted by shRNAs with a resultant epidermal remodeling, but that only TNFα knockdown results in a clinical improvement of the psoriatic phenotype within the experimental time frame. It should be kept in mind that the clinical observation is only a superficial score, and cannot reflect the disease status in the deeper layers. The large variations in the semiquantitative clinical psoriasis scores also confirm this. E.g. a scale that has yet not sloughed off may hide a more alleviated disease state of the skin, but is not registered in the clinical score. More emphasis must therefore be given to the epidermal thickness measurements as this measure gives a more in-depth evaluation of the disease.

TNFα mRNA knockdown has a significant positive effect on the clinical psoriatic phenotype throughout the treatment course, whereas the effect of IL-12B mRNA knockdown in the current study is minimal. Such difference between targeting of IL-12B and of TNFα could hypothetically be explained by the different functions of the two cytokines. If IL-12 and IL-23 are less potent immune mediators than TNFα is, then TNFα knockdown may have a more profound therapeutic effect on psoriasis. The cytokines may also differ in abundance and redundancy, as well as the dynamics and kinetics of their immune signaling during inflammation. The cellular source of the aberrant expression of TNFα and IL-12B is somewhat similar [43]. Therefore, although a difference in transduction efficiency of the various cellular constituents of the skin could influence the response to different cytokine-directed shRNAs, this may not necessarily explain our findings. Alternatively, the variation between cytokine targets may reflect differences in cytokine mRNA expression levels and, thus, the knockdown efficiencies that are required to obtain a therapeutic benefit. Although systemic treatment with anti-p40 monoclonal antibodies resulted in robust clinical improvement in the xenotransplantation model, we cannot exclude the possibility that a local RNA-directed treatment targeting IL-12B would require a longer time-frame (> 3 weeks to obtain a therapeutic effect and improve the disease phenotype.

The skin offers an attractive organ for RNAi-based therapeutics due to its ease of access. A therapeutic approach relying on lentiviral vector-mediated delivery of shRNAs to the skin is far from clinically relevant due to safety issues concerning the vector. So far, topical non-viral delivery of RNAi effectors have been hampered by poor penetrability of the stratum corneum, but recent advances have made it possible to deliver siRNAs formulated in a cream to the epidermis and dermis of murine skin at therapeutically relevant levels [44]. Even though topical local delivery of siRNAs does not mediate a sustained RNAi effect, it has the potential to overcome multiple problems regarding systemic biological therapeutics including complications concerning patient-friendly administration, contra-indications, side-effects and patient adherence. Based on our current knowledge, we suggest that siRNAs targeting TNFα rather than IL-12B are further explored for clinical use.

## Conclusion

Our studies show potent and sustained RNAi-mediated down-regulation of IL-12B mRNA by the use of lentiviral vectors. We document efficient lentiviral-mediated gene delivery and persistent gene expression in xenografted human skin. Down-regulation of IL-12B mRNA in xenografted psoriatic skin leads to a decrease in epidermal thickness, but not a clinical amelioration of the psoriatic phenotype in contrast to our previous studies targeting TNFα mRNA. Our studies question IL-12B mRNA as an optimal target for RNAi-directed treatment and, hence, further strengthen the need for validating therapeutic targets for such treatment. Small RNAs that mediate a cytokine-targeting RNAi response or interfere with endogenous cytokine regulation have the potential to become novel anti-inflammatory therapeutics, but additional obstacles such as off-targeting effects and poor delivery must be addressed before small RNAs can prove their therapeutic worth.

## Competing interests

The authors declare that they have no competing interests.

## Authors' contributions

ROB carried out shRNA design, shRNA oligonucleotide cloning, cellular *in vitro *experiments including luciferase assays, production of lentiviral vectors and qRT-PCRs. Additionally ROB wrote the manuscript. KS and CR carried out xenotransplantation and treatments of all skin grafts as well as assessment of semiquantitative clinical psoriasis scores, measurements of epidermal thicknesses, and immunohistochemical stainings. Furthermore, KS and CR participated in designing the study and helped draft the manuscript. LBP was in charge of measurements of bioluminescence with the assistance of FDH. The lentiviral vectors pCCL-PGK-Puro-H1-MCS and pLV/PGK-FLuc were constructed by BM and MJ, respectively. SK and TND obtained psoriatic skin samples from patients. TGJ participated in the design of the study. JGM played a crucial role in the design and coordination of the study and wrote the manuscript together with ROB. All authors read and approved the final manuscript.

## Pre-publication history

The pre-publication history for this paper can be accessed here:

http://www.biomedcentral.com/1471-5945/11/5/prepub
